# Synthesis and characterization of crystalline polymeric carbonic acid (H_2_CO_3_) with *sp*^3^-hybridized carbon at elevated pressures

**DOI:** 10.1038/s42004-025-01614-y

**Published:** 2025-08-08

**Authors:** Dominik Spahr, Lkhamsuren Bayarjargal, Lukas Brüning, Valentin Kovalev, Lena M. Wedek, Maxim Bykov, Victor Milman, Nico Giordano, Björn Winkler, Elena Bykova

**Affiliations:** 1 Institute of Geosciences, Goethe University Frankfurt, Frankfurt, Germany; 2 Institute of Inorganic and Analytical Chemistry, Goethe University Frankfurt, Frankfurt, Germany; 3 Dassault Systèmes BIOVIA, Cambridge, UK; 4 Deutsches Elektronen-Synchrotron DESY, Hamburg, Germany

**Keywords:** Inorganic chemistry, X-ray diffraction

## Abstract

The existence of polymeric carbonic acid (H_2_CO_3_) at elevated pressures has been predicted, but has not been investigated experimentally. Here, polymeric carbonic acid containing *sp*^3^-hybridized carbon was synthesized and characterized at  ≈40 GPa. H_2_CO_3_ single crystals were obtained by laser-heating a H_2_O + CO_2_ mixture in a diamond anvil cell. The orthorhombic crystal structure (*Cmc*2_1_ with *Z* = 4) was refined from synchrotron single crystal X-ray diffraction data and is in agreement with a structural model predicted earlier. The crystal structure of H_2_CO_3_-*Cmc*2_1_ is characterized by ${[{{\rm{CO}}}_{4}]}^{4-}$ building blocks which are connected via corner sharing, forming chains along the *c*-axis. The combination of single crystal X-ray data with experimental Raman spectroscopy and DFT-calculations confirms that the structural model of H_2_CO_3_-*Cmc*2_1_ is appropriate. The synthesis condition of polymeric carbonic acid points towards its potential existence in ice giants including the ones present in our solar system.

## Abstract (web summary)

Crystal structure predictions have proposed the existence of polymeric H2CO3, but its high-pressure polymorphism has yet to be confirmed experimentally. Here, the authors synthesized single crystals of polymeric H_2_CO_3_ in a diamond anvil cell and demonstrated that its structure consists of polymerized ${[{{\rm{CO}}}_{4}]}^{4-}$ tetrahedra forming chains along the c-axis.

## Introduction

Carbonic acid (H_2_CO_3_) is an extensively studied, but still enigmatic, compound. It is well established that H_2_CO_3_ forms in small quantities by the reaction between H_2_O and CO_2_ in aqueous solutions, but either decomposes back or rapidly dissociates^1,2^. This poses significant experimental challenges for the crystallization of H_2_CO_3_, which is a prerequisite for single-crystal structure determinations. Numerous approaches have been employed to overcome this hurdle, including particle or light irradiation of H_2_O-ice:CO_2_ mixtures and synthesis at high-pressures^3–9^.

**Fig. 1:**
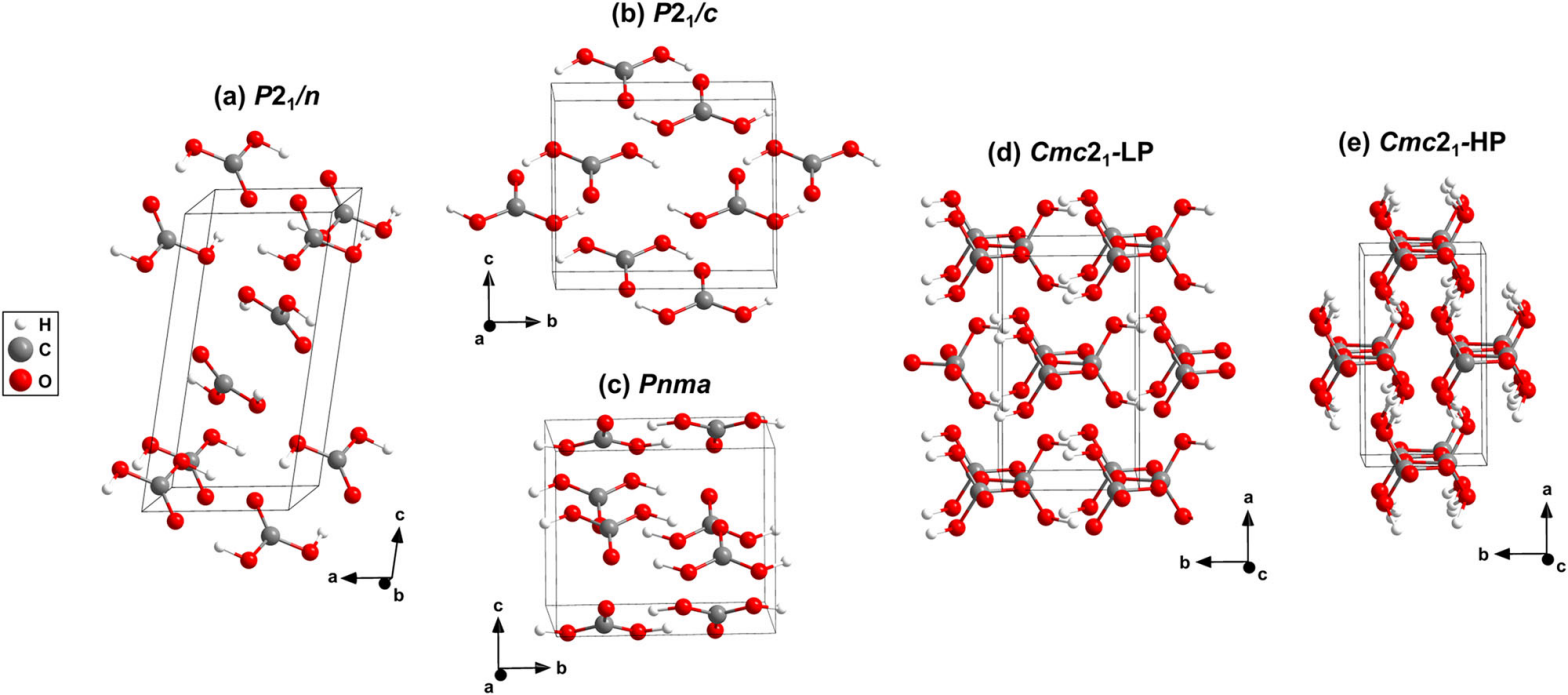
Experimental and calculated crystal structures of the H_2_CO_3_ phases. **a** Experimental crystal structure of H_2_CO_3_-*P*2_1_/*n* (≈8 GPa) from single crystal X-ray diffraction^8^. **b** Experimental crystal structure of deuterated H_2_CO_3_-*P*2_1_/*c* (≈2 GPa)^5^. Predicted crystal structures of: **c** H_2_CO_3_-*Pnma* (1 GPa)^6^, **d** the low-pressure phase of H_2_CO_3_-*Cmc*2_1_ (100 GPa)^6^ and **e** the high-pressure phase of H_2_CO_3_-*Cmc*2_1_(400 GPa)^6^.

Due to the experimental challenges, a number of problematic results were obtained. For example, the conclusions regarding the existence of *α*-H_2_CO_3_, based on spectroscopic evidence, needed to be revised, as it was later shown that in fact the monomethyl ester of H_2_CO_3_, (CH_3_OCO_2_H) had been obtained^10–13^. This led to the conclusion that *β*-H_2_CO_3_ was the only ambient pressure phase, but its exact crystal structure remained unknown^13^. More recently, there were attempts to combine density functional theory (DFT)-based calculations and high-pressure studies to obtain and confirm structural models for crystalline H_2_CO_3_. However, a recent neutron powder diffraction study on a deuterated sample, which yielded a low pressure (≈2 GPa) monoclinic structure, H_2_CO_3_-*P*2_1_/*c* (Fig. 1b), is problematic, as it is evident from the published data that the amount of H_2_CO_3_ in the sample was minute in comparison with the co-existing CO_2_-I phase (dry ice), and the refinements relied on constraints and restraints^5^.

Earlier, a crystal structure prediction study had been published in which several polymorphs of H_2_CO_3_ were proposed^6^. Specifically, for the pressure range of 1–44 GPa, a phase with space group *Pnma* was predicted (Fig. 1c). For the pressure range from 44 to 314 GPa, it was predicted that carbon would be *sp*^3^-hybridized, and that the ${[{{\rm{CO}}}_{4}]}^{4-}$-tetrahedra would polymerize to form chains in two orthorhombic structures both having space group *Cmc*2_1_ (Fig. 1d, e)^6^. A phase transition between two H_2_CO_3_-*Cmc*2_1_ polymorphs was predicted to occure at 240 GPa. The high pressure phase of H_2_CO_3_-*Cmc*2_1_ was predicted to remain stable up to its reaction with H_2_O to orthocarbonic acid (H_4_CO_4_) above 314 GPa^6^. It was proposed that the computed vibrational spectra for the low pressure phase H_2_CO_3_-*Pnma* were in agreement with the experimentally obtained IR and Raman spectra measured at  ≈4 GPa in a study reported earlier^6,7^.

Recently, we provided the first X-ray single crystal structure solution of water-free H_2_CO_3_ at low pressures (≈8 GPa) with *P*2_1_/*n* space group symmetry (Fig. 1a)^8^. The geometry of the H_2_CO_3_ molecules in *cis-cis* configuration is in reasonable agreement with their geometry in the chemically closely related carbonic acid monohydrate (H_2_CO_3_. H_2_O), which has also been studied by single crystal X-ray diffraction (at 6.5 GPa) in earlier experiments^9^. We complemented our diffraction study by a combination of experimental Raman spectroscopy and DFT calculations.

A single crystal structure determination, with a high reflection-to-parameter ratio (>8) and low *R*-values (a few %), is the gold standard for establishing new structures, even if reciprocal space has not been fully explored, as is typical in DAC-based studies. This is especially the case if the structure is then reproduced in full geometry optimizations in DFT calculations and the latter reproduce the Raman spectrum. This implies that not only the structure correspond to a local energy minimum, but also that the second and third derivatives of the energy hypersurface have been obtained correctly. There is no example of a moderately complex structure where the experimental Raman spectrum has been reproduced by DFT calculations for a wrong structure, and hence the crystal structure of H_2_CO_3_-*P*2_1_/*n* at pressures of 5–13 GPa is now established^8^. Its crystal structure is distinct from H_2_CO_3_-*Pnma* or H_2_CO_3_-*P*2_1_/*c*^5,6^. In H_2_CO_3_-*P*2_1_/*n* carbon is *sp*^2^-hybridized, i.e., this is a “conventional” carbonate, characterized by nearly trigonal ${[{{\rm{CO}}}_{3}]}^{2-}$-groups. Two H_2_CO_3_ molecules in *cis-cis* geometry are connected by two hydrogen bonds forming layers. This crystal structure had not been predicted in the study by Saleh and Oganov^6^.

Due to the disagreement between the crystal structure prediction approach for the low-pressure phase of H_2_CO_3_ and the experimental data in this pressure range and the absence of any experimental high pressure structural studies, the high pressure polymorphism of H_2_CO_3_ is unresolved. Determining the high pressure polymorphism of H_2_CO_3_ is a relevant effort for several reasons. From a crystallographic and crystal chemical point of view, the crystal structure prediction of Saleh and Oganov^6^ is exciting, as the polymorph predicted to be stable at pressures between 44 and 314 GPa was thought to contain polymerized ${[{{\rm{CO}}}_{4}]}^{4-}$-groups forming chains. The polymerization of ${[{{\rm{CO}}}_{4}]}^{4-}$-groups in carbonates has been well established for more than a decade now. However, pressure-induced polymorphic transitions from *sp*^2^- to *sp*^3^-carbonates occur only at much higher pressures (e.g., at  ≈85 GPa for MgCO_3_, at  ≈105 GPa for CaCO_3_, and at  ≈115 GPa for Ca_1.5_Fe_0.9_Mg_0.6_C_3_O_9_)^14–16^. In contrast, *sp*^3^-carbonates can be obtained at much lower pressures if instead of pressure induced polymorphic transitions reactions with a corresponding oxide (e.g., at  ≈20 GPa for Sr_2_CO_4_) or with CO_2_ (e.g., at  ≈34 GPa for Ca_2_C_4_O_1_0) are induced^17,18^. In carbonates, the polymerization of ${[{{\rm{CO}}}_{4}]}^{4-}$-groups into infinite chains is very rare and has been observed only in CaCO_3_-*P*2_1_/*c* at  ≈105 GPa^15^.

Solid H_2_CO_3_ is also relevant for astrophysical studies. H_2_O and CO_2_ are components of interstellar dust, and very large stellar objects exist which contain enormous amounts of H_2_O and CO_2_^19^. Furthermore, H_2_O and CO_2_ are present in the frozen nuclei of comets and on/in icy bodies in our solar system^20–25^. High pressures up to  ≈1000 GPa and temperatures up to several thousand Kelvin in combination with the presence of large H_2_O-ice containing shells have been found in ice giants such as Uranus and Neptune^26,27^. As CO_2_ is present in their atmosphere^28^, it is possible that high pressure polymorphs of H_2_CO_3_ form inside of ice giants. Hence, an understanding of the system H_2_O–CO_2_ as a function of pressure and temperature is required to establish spectral libraries for their remote identification. The relevance of H_2_CO_3_ in our solar system is also evident from the recent spectroscopic evidence of its presence on the surface of Ganymede reported by the Jovian Infrared Auroral Mapper (JIRAM) or by the detection of CO_2_ together with H_2_O_2_ on the stratified surface of Charon using the James Webb Space Telescope (JWST)^29,30^. Furthermore, the JWST detected H_2_O together with CO_2_ in the atmosphere of the hot Super-Neptune WASP-166b^31^.

## Results and discussion

The high-pressure experiments on the H_2_O-CO_2_ system were carried out in laser-heated diamond anvil cells (LH-DACs). In a first step, a drop of H_2_O was added into the sample chamber of a DAC. A significant amount of water was then allowed to evaporate before closing the DAC tightly. In the next step, the DAC was cooled down. After reaching  ≈273 K the DAC was reopened before being cooled further to  ≈100 K. At this temperature dry ice was directly condensed into the sample chamber from a CO_2_ gas jet. The DAC was tightly closed after the sample chamber was completely covered with dry ice and then compressed to the target pressure of the experiment without intermediate heating. The sample pressure during compression was derived from the position of the high frequency edge of the diamond Raman band^32^. After tightly closing the DAC the pressure in the sample chamber was  ≈10 GPa. At this pressure, CO_2_-I ($Pa\bar{3}$) is the stable phase up to its melting temperature^33,34^. In a Raman experimental spectrum, obtained after the cryogenic loading, we could identify the characteristic Raman modes of CO_2_-I (Fig. S2a) at low wavenumbers (<300 cm^−1^)^35^. In addition, we observed the characteristic Raman signal of H_2_O-VII at high wavenumbers (≈3000–3400 cm^−1^) (Fig. S2b), confirming that H_2_O is present in the sample chamber^36,37^.

**Fig. 2:**
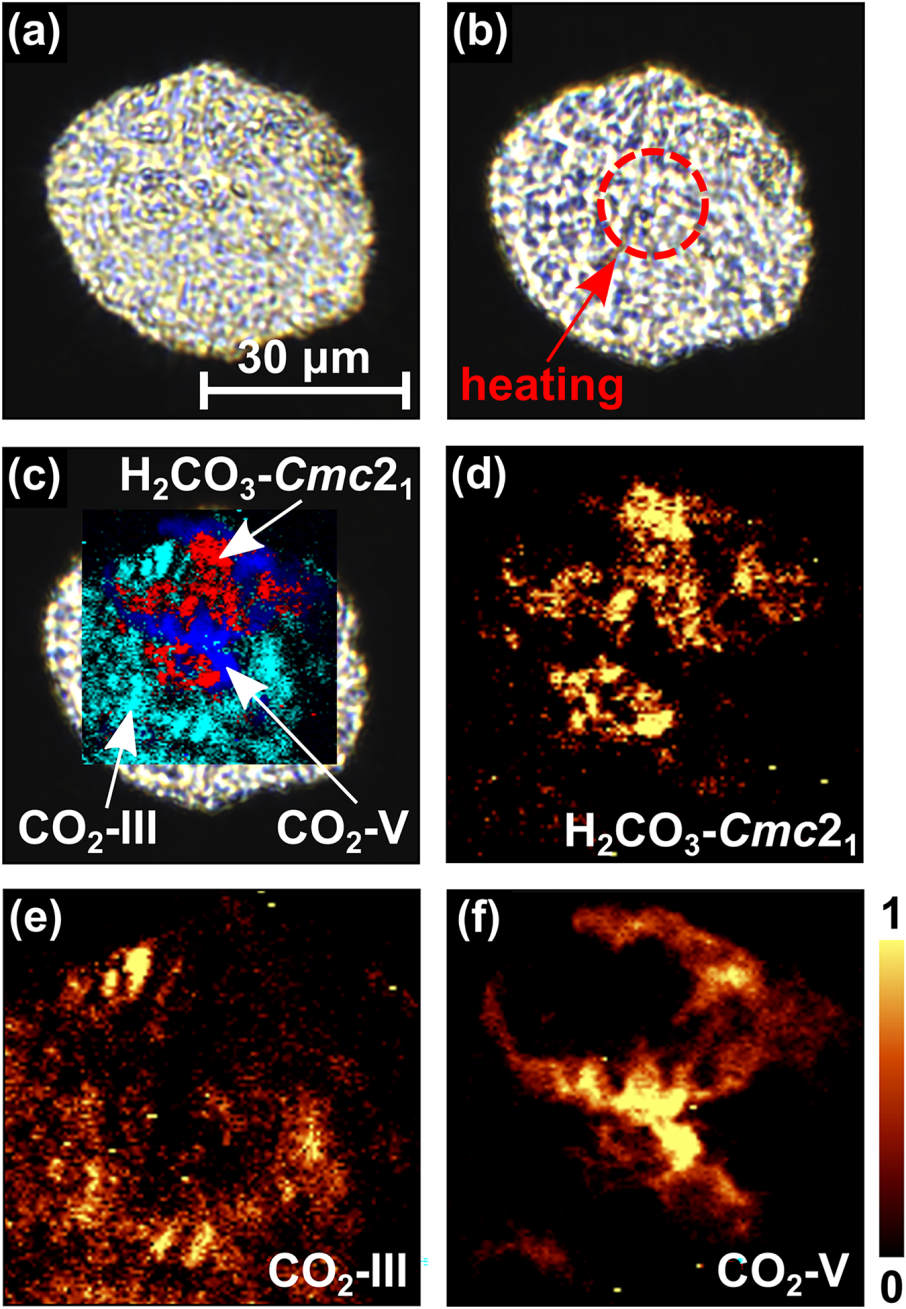
Sample chamber of the DAC and Raman maps of H_2_CO_3_ and CO_2_ after the synthesis. **a** Sample chamber of the DAC with the H_2_O + CO_2_ mixture before the laser heating at 40(2) GPa. **b** Sample chamber after heating the mixture up to a maximum temperature of  ≈1000 K. **c** 2D-Raman map showing the distribution of H_2_CO_3_-*Cmc*2_1_, CO_2_-III, and CO_2_-V overlayed on a picture of the sample chamber. Raman map of: **d** H_2_CO_3_-*Cmc*2_1_ (≈910 cm^−1^), **e** CO_2_-III (≈380 cm^−1^) CO_2_-V (≈810 cm^−1^), and **f** CO_2_-V (≈810 cm^−1^) after laser heating at 40(2) GPa.

**Fig. 3:**
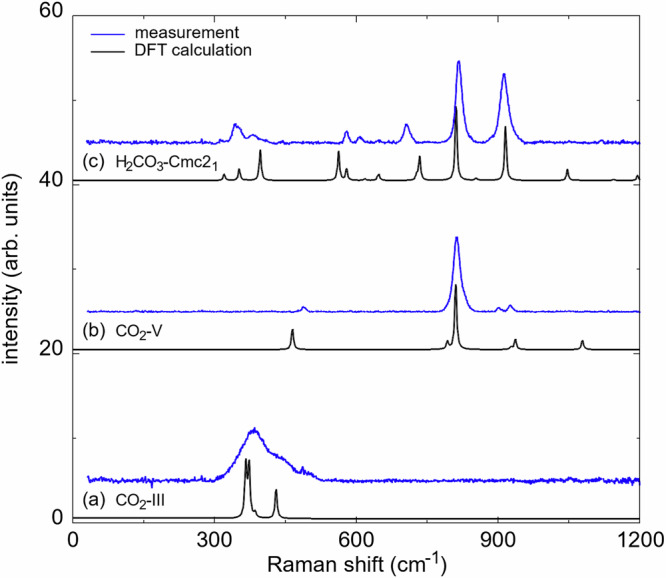
Experimental and calculated Raman spectra before and after the synthesis of H_2_CO_3_–*Cmc*2_1_ at 40(2) GPa. **a** Raman spectra for the untransformed low-pressure phase CO_2_-III. **b** Raman spectra for the high-pressure polymorph CO_2_-V. **c** Raman spectra of H_2_CO_3_-*Cmc*2_1_ after the synthesis. Experimental Raman spectra (blue lines) were collected in the same DAC corresponding to the 2D-Raman maps in Fig. 2c–f. DFT-based calculations (black lines) were rescaled by 1–3%.

Figure 2 a shows the sample chamber of the DAC after cold-compression of the CO_2_ + H_2_O mixture to 40(2) GPa before laser heating. During compression a phase transition from CO_2_-I ($Pa\bar{3}$) to CO_2_-III (*Cmca*) occurs in a broad (≈5 GPa) pressure interval around  ≈12 GPa^33,35^. The experimental Raman data of unheated CO_2_-III at 40(2) GPa are in good agreement with the Raman spectrum derived from our DFT-based calculations (Table S3) in space group *Cmca* (Fig. 3a). At 40 GPa the stable phase of water is H_2_O-VII ($Pn\bar{3}m$)^38,39^, but due to the overtone of the diamond the characteristic Raman modes of H_2_O-VII at high wavenumbers cannot be observed at this pressure^36^.

At the target pressure of the experiment (40(2) GPa) we used double-sided CO_2_ laser-heating to induce the reaction. The sample was heated to a maximum temperature of  ≈1000(300) K in one spot of the sample chamber (Fig. 2b). At this pressure, the direct and indirect heating of CO_2_-III causes the appearance of the high-pressure polymorph CO_2_-V in several parts of the sample chamber (Fig. 2f), which is the stable polymorph of CO_2_ to pressures above 100 GPa.^34,40^. CO_2_-III is still present in some unheated regions of the sample chamber (Fig. 2e). The experimental Raman spectra of CO_2_-V show a strong characteristic Raman mode at  ≈810 cm^−1^ and are in very good agreement with our calculated Raman spectra (Table S4) in space group $I\bar{4}2d$ (Fig. 3b). In addition, we found that laser heating at 40(2) GPa causes the formation of a phase with a strong new Raman mode at  ≈910 cm^−1^ (Fig. 3c). Strong Raman modes of carbonates occurring at such low wavenumbers are typically present in *sp*^3^-carbonates such as Sr_2_CO_4_ or Ca_2_CO_4_ and characteristic for the C–O stretching mode in a ${[{{\rm{CO}}}_{4}]}^{4-}$-group^17,18^. From spatially resolved Raman spectroscopy we found that the distribution of the unknown phase in the sample chamber is not identical with the one of CO_2_-V (Fig. 2d, f).

**Fig. 4:**
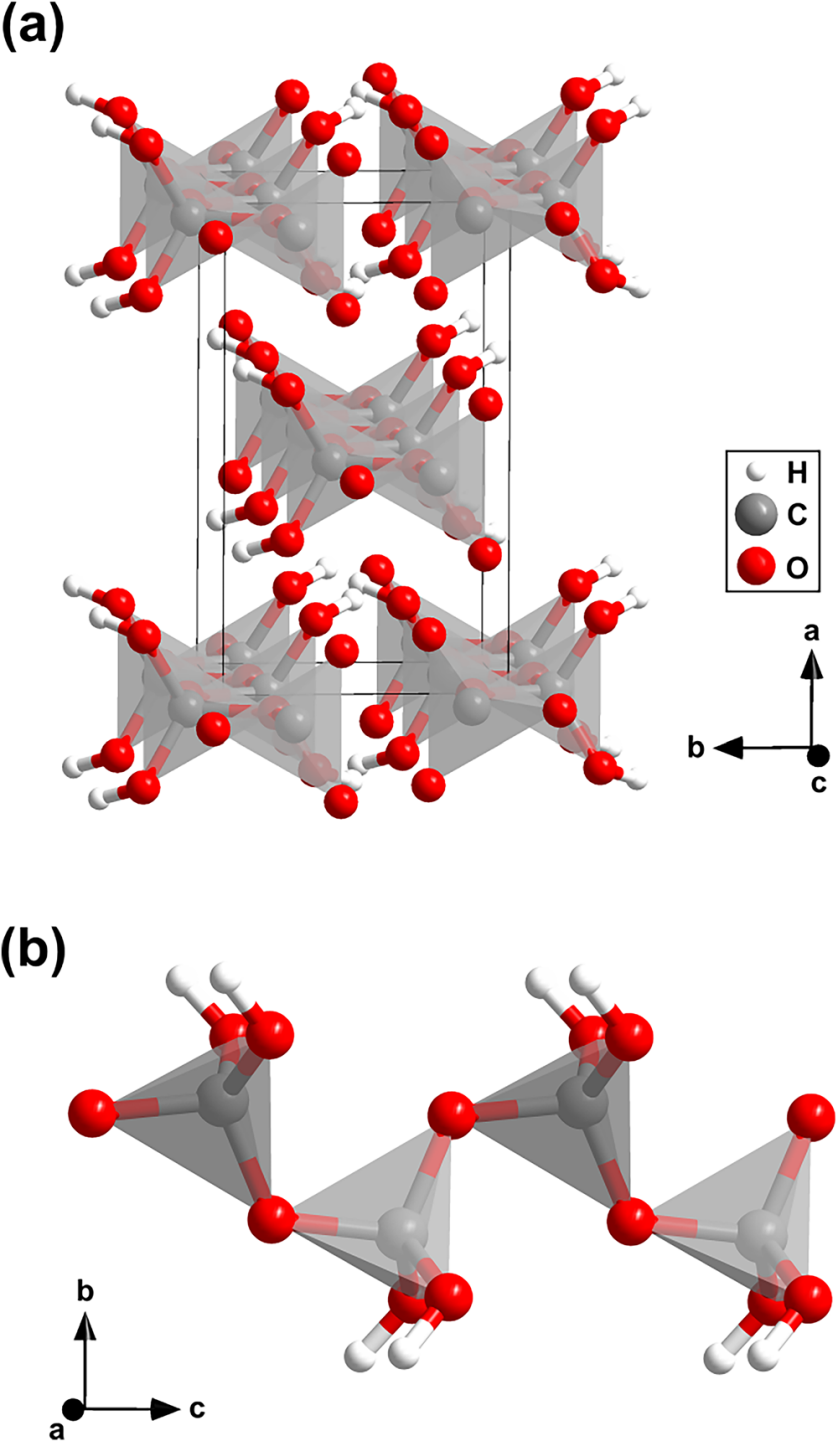
Crystal structure of H_2_CO_3_-*Cmc*2_1_ and polymerized protonated ${[{{\rm{CO}}}_{4}]}^{4-}$-tetrahedra. **a** Orthorhombic crystal structure (*Cmc*2_1_, *Z* = 4) of polymeric carbonic acid (H_2_CO_3_) at 40(2) GPa from single crystal structure refinement. **b** Polymerized protonated ${[{{\rm{CO}}}_{4}]}^{4-}$-tetrahedra in the crystal structure of H_2_CO_3_-*Cmc*2_1_ forming chains along the *c*-axes.

We obtained synchrotron X-ray diffraction patterns on regularly spaced grid points across the sample chamber in order to locate promising positions for the subsequent collection of single crystal diffraction data. We collected diffraction data suitable for single crystal X-ray diffraction analysis using a  ≈ 2 × 2 μm^2^-sized X-ray beam at selected locations (see SI). From these data we solved the crystal structure of the new phase and found that it is orthorhombic H_2_CO_3_-*Cmc*2_1_ with *Z* = 4 (Fig. 4a).

This structure had been predicted by evolutionary algorithm in the C–H–O ternary system (Fig. 1c), where a stability field of 44–240 GPa was proposed^6^. The lattice parameters from single crystal structure data analysis at 40(2) GPa (Fig. 4a) are *a* = 7.286(3) Å, *b* = 4.275(1) Å and *c* = 3.809(4) Å (*V* = 118.6(1) Å^3^). The relatively low *R*_1_-value of 4.6% for a DAC experiment is indicative of a reliable structure refinement (see SI). Our DFT-based full geometry optimizations accurately reproduce the experimentally determined structure and earlier predictions (Table S1)^6^. Furthermore, our experimental Raman spectrum is in good agreement with the theoretical one derived from our DFPT-based calculations (Fig. 3c, Table S5), providing further confirmation that the structural model of H_2_CO_3_-*Cmc*2_1_ is appropriate at elevated pressures.

The crystal structure is characterized by polymerized ${[{{\rm{CO}}}_{4}]}^{4-}$-tetrahedra forming infinite chains along the *c*-axes (Fig. 4b). The single crystal structure determination of H_2_CO_3_-*Cmc*2_1_ is the first experimental proof for the polymerization of *sp*^3^-hybridized ${[{{\rm{CO}}}_{4}]}^{2-}$-groups in carbonic acid. Each of the ${[{{\rm{CO}}}_{4}]}^{4-}$-tetrahedra is protonated by two hydrogen atoms. The location of hydrogen positions is often assumed to be experimentally challenging in high-pressure diffraction experiments. However, we demonstrated earlier that in favorable circumstances, i.e., the absence of strongly scattering heavy elements, the hydrogen positions can be reliably located with our experimental approach^8,41^. Nevertheless, as in all X-ray diffraction experiments, an accurate determination of the O–H distance is often not possible, and so, in order to reduce the error associated with the bond distance, we introduced a restraint for the O–H bond distance in our experimental model (≈0.9 Å).

**Fig. 5:**
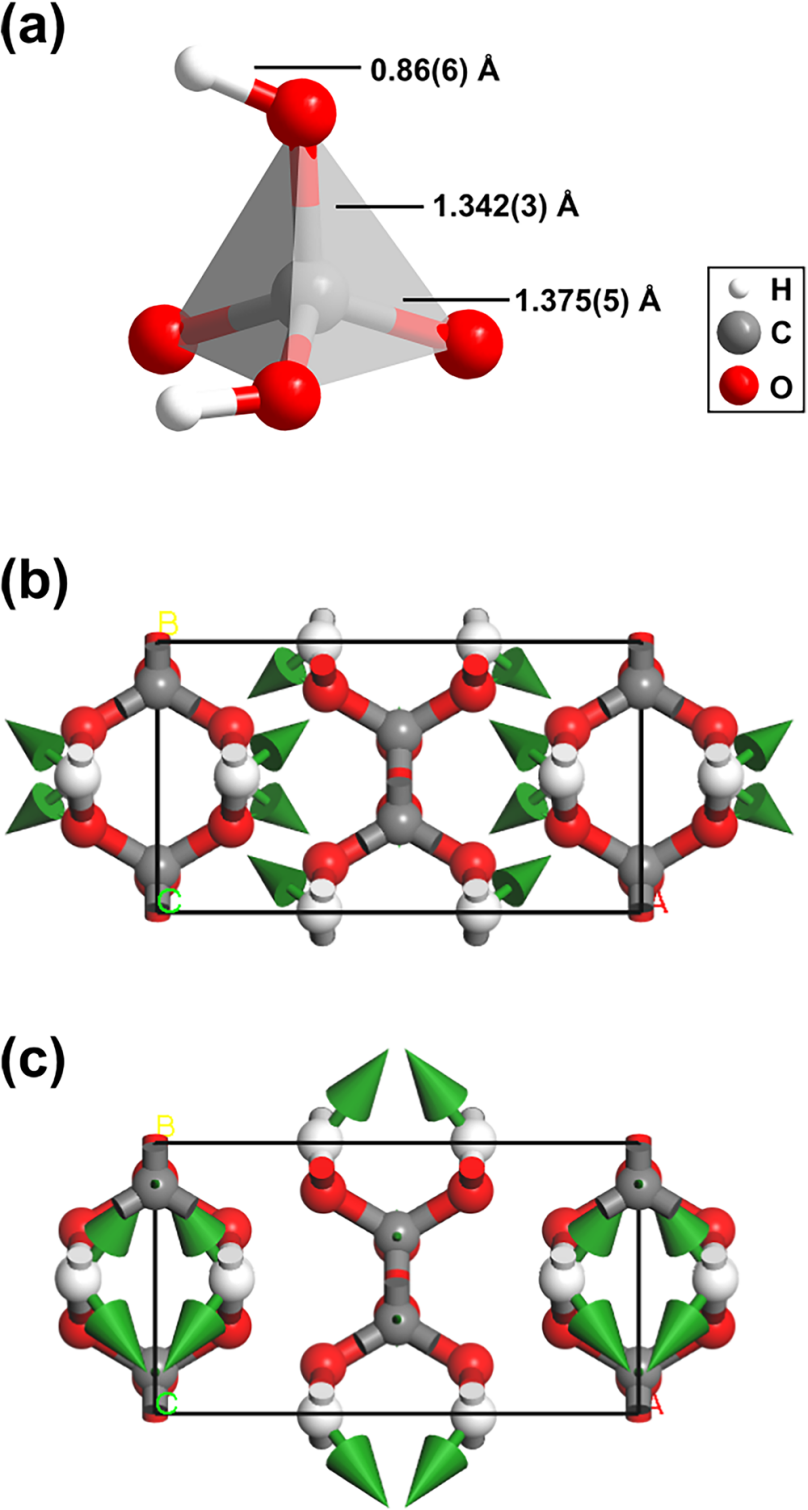
Geometry of a protonated ${[{{\rm{CO}}}_{4}]}^{4-}$-tetrahedron and eigenvectors of the atomic displacements of selected characteristic Raman modes. **a** Geometry of one protonated ${[{{\rm{CO}}}_{4}]}^{4-}$-tetrahedron in the crystal structure of H_2_CO_3_-*Cmc*2_1_ from single crystal structure refinement at 40(2) GPa. Eigenvector of the atomic displacements in polymeric carbonic acid for the characteristic Raman mode **b** at  ≈820 cm^−1^ and **c** at  ≈910 cm^−1^ from DFPT calculations.

At 40 GPa two of the four C–O bond distances within one ${[{{\rm{CO}}}_{4}]}^{4-}$-tetrahedron (Fig. 5a) are identical (1.342(3) Å) due to symmetry constraints, while the other two are slightly longer (1.375(5) Å/1.370(9) Å). The DFT model predicts C–O bond lengths between 1.352 and 1.399 Å. The Mulliken population of the C–O bonds decrease from 0.66 to 0.55 e^−^/Å^3^ with the C–O distance. These values are indicative of predominantly covalent bonds. Due to the polymerization of the ${[{{\rm{CO}}}_{3}]}^{2-}$-groups in H_2_CO_3_-*Cmc*2_1_ to ${[{{\rm{CO}}}_{4}]}^{4-}$ tetrahedra, the *sp*^3^-hybridized C–O bond distances are significantly longer in H_2_CO_3_-*Cmc*2_1_ than in the *sp*^2^-hybridized ${[{{\rm{CO}}}_{3}]}^{2-}$-groups of hypothetical H_2_CO_3_-*P*2_1_/*n* at 40 GPa (1.25–1.28 Å)^8^. The increase of the C–O bond length (≈7%) is in good agreement with other *sp*^2^–*sp*^3^ phase transitions. For example, Ca[CO_3_]-*P*2_1_/*c* undergoes a *sp*^2^–*sp*^3^ transition at 105 GPa, which is accompanied by an increase of the C–O bond distances from 1.228 to 1.315 Å (≈7%)^15^. In the DFT model the covalent O–H bond is 1.016 Å long and has a Mulliken bond population of 0.53 e^−^/Å^3^, in contrast to the O⋯H bond which is 1.455 Å long and has a population of 0.17 e^−^/Å^3^. At 40 GPa, the hydrogen bonds are slightly kinked (∢ O–H⋯O = 163^∘^) and the O–H⋯O distance is 2.444 Å indicating the presence of very strong hydrogen bonds^42^.

We carried out DFPT calculations in order to calculate eigenvectors for selected vibration. Figure 5b, c shows the displacements in the H_2_CO_3_ molecule for the characteristic Raman modes at  ≈820 cm^−1^ and at  ≈910 cm^−1^. Both modes are due to a displacement of the hydrogen atoms only, where in the  ≈820 cm^−1^ the hydrogen moves perpendicular to the covalent OH-bond, while in the  ≈910 cm^−1^ there is also a substantial stretching contribution. The strong Raman mode at  ≈820 cm^−1^ is close to a Raman mode of CO_2_-V, but a closer inspection of the peak positions of the experimental Raman data showed that the Raman mode of CO_2_-V is at  ≈810 cm^−1^ while the one of H_2_CO_3_-*Cmc*2_1_ is at  ≈820 cm^−1^. This difference in shifts can be resolved experimentally and Raman maps show the strongest intensity of the two phases at different locations in the sample chamber (Fig. 2c–f).

We used our DFT-calculations to obtain the bulk modulus for H_2_CO_3_-*Cmc*2_1_. We fitted an equation of state (EoS) to the *p*, *V* relation derived from the calculations (Fig. S4) and obtained a bulk modulus of *K*_0_ = 50(2) GPa with *K*_p_ = 5.2(1). As expected, this is significantly higher than the one obtained for the unpolymerized, low-pressure *sp*^2^-polymorph H_2_CO_3_-*P*2_1_/*n* (*K*_0_ = 14.2(4) GPa with *K*_p_ = 6.1(1))^8^. The bulk modulus of H_2_CO_3_-*Cmc*2_1_ is between the relatively high bulk modulus of polymeric CO_2_-V (*K*_0_ = 136(11) GPa with *K*_p_ = 3.7(4)) and the significantly lower bulk modulus of H_2_O-VII (*K*_0_ = 23.9(7) GPa with *K*_p_ = 4.2(5))^40,43^. We found that the compression behavior of H_2_CO_3_-*Cmc*2_1_ is significantly anisotropic since strong hydrogen bonds are located at the *b*, *c*-plane (Fig. S5). Between 30 and 90 GPa, the compression along the *a*-axis is  ≈10%, while it is only  ≈6% along the *b*- and *c*-axes in the same pressure range.

## Conclusion

In summary, we have synthesized and characterized single crystals of a high pressure polymorph of carbonic acid H_2_CO_3_-*Cmc*2_1_, obtained by a reaction between H_2_O and CO_2_ at elevated pressures and temperatures (≈40 GPa and  ≈1000 K). This polymorph is characterized by polymerized ${[{{\rm{CO}}}_{4}]}^{4-}$-tetrahedra, which form chains along the *c*-axis by corner-sharing. The experimental crystal structure of H_2_CO_3_-*Cmc*2_1_ confirms the model predicted earlier based on an evolutionary algorithm^6^. In addition to the experimental description of the crystal structure, we calculated the *p*, *V*-relation and derived parameters of the H_2_CO_3_-*Cmc*2_1_ EoS. Moreover, we have provided an experimental and calculated Raman spectrum for this compound, which will facilitate its identification in future experiments.

Our results significantly advance the understanding of the crystal chemistry of carbonates at high pressures, as this is only the second system, after CaCO_3_^15^, which is known to form linear infinite chains by corner sharing of ${[{{\rm{CO}}}_{4}]}^{4-}$-tetrahedra. H_2_CO_3_-*Cmc*2_1_ is a reference point in high-pressure research to study the influence of the hydrogen bonding on the formation conditions and crystal structures of hydrous *sp*^3^-carbonates. While the detailed examination of the stability field of H_2_CO_3_-*Cmc*2_1_ was outside the scope of the current study, our experimental data suggest that H_2_CO_3_-*Cmc*2_1_ may occur inside the H_2_O-rich ice shells in ice giants such as Uranus or Neptune, making it relevant for astrophysical research. This is supported by the presence of H_2_O and CO_2_ in the atmosphere of the hot Super-Neptune WASP-166b^31^.

## Methods

The high-pressure experiments were carried out in Boehler-Almax type diamond anvil cells (DACs)^44^. For the loading of the DACs, we used bidistilled water obained from a GFL Glass Bi-Distiller and CO_2_-gas as purchased (Nippon gases, purity ≥99.995%). CO_2_ was loaded as dry ice by cryogenic loading into the DAC using a custom-built cryogenic loading system (see Spahr et al.^45^), derived from an earlier concept developed for similar studies^46^. At the target pressure of the experiment laser-heating was performed from both sides using a custom-built set-up equipped with a Coherent Diamond K-250 pulsed CO_2_ laser (*λ* = 10,600 nm)^47^. Raman spectroscopy was performed using an Oxford Instruments WITec alpha 300R Raman imaging microscope equipped with an Olympus SLMPan N 50× objective. The measurements were performed using the 532 nm laser and the 1800 grooves mm^−1^ grating of the WITec UHTS 300S (VIS-NIR) spectrograph. Single-crystal synchrotron X-ray diffraction was carried out at the synchrotron PETRA III (DESY) in Hamburg, Germany, at the Extreme Conditions Beamline P02.2^48^. The beam size on the sample was  ≈2 × 2 μm^2^ (FWHM) using a wavelength of 0.2903 Å (42.7 keV). The diffraction data were collected using a Perkin Elmer XRD1621 detector. First-principles calculations were carried out within the framework of DFT, employing the Perdew-Burke-Ernzerhof (PBE) exchange-correlation functional and the plane wave/pseudopotential approach implemented in the CASTEP simulation package^49–51^. We employed the correction scheme for van der Waals (v.d.W.) interactions developed by Tkatchenko and Scheffler^52^. A detailed description of the experimental and computational methods is available in the supplementary material.

## Supplementary information

Supplementary Material

## Data Availability

The X-ray crystallographic coordinates for the structure reported in this study has been deposited at the Cambridge Crystallographic Data Centre (CCDC), under deposition numbers 2420110 (single crystal) and 2420111 (DFT calculation). These data can be obtained free of charge from The Cambridge Crystallographic Data Centre via www.ccdc.cam.ac.uk/data_request/cif. The supplementary material contains additional information to the results of the single crystal structure determination, Raman spectroscopy, and DFT-based calculations.
