# Supplementary material for: Synthesis and characterization of crystalline polymeric carbonic acid (H2CO3) with sp3-hybridized carbon at elevated pressures
Source: Commun Chem. 2025 Aug 8;8:237. doi: 10.1038/s42004-025-01614-y (PMC12334750; doi:10.1038/s42004-025-01614-y)
Supplement: Supplementary file 1 — Supplementary Material [file 42004_2025_1614_MOESM1_ESM.pdf]

Supplementary material: Synthesis and Characterization of Polymeric Carbonic Acid (H_2CO_3) with sp^3-Hybridized Carbon at Elevated Pressures

Dominik Spahr[*,a], Lkhamsuren Bayarjargal[a], Lukas Brüning[b], Valentin Kovalev[a], Lena M. Wedek[a], Maxim Bykov[b], Victor Milman[c], Nico Giordano[d], Björn Winkler[a], Elena Bykova[a]

[a]*Goethe University Frankfurt, Institute of Geosciences, Altenhöferallee 1, 60438 Frankfurt, Germany*
[b]*Goethe University Frankfurt, Institute of Inorganic and Analytical Chemistry, Max-von-Laue-Straße 7, 60438 Frankfurt, Germany*
[c]*Dassault Systèmes BIOVIA, 334 Cambridge Science Park, Cambridge CB4 0WN, United Kingdom*
[d]*Deutsches Elektronen-Synchrotron DESY, Notkestrasse 85, 22607 Hamburg, Germany*

1. Supplementary Methods

1.1. Sample material

For the loading of the diamond anvil cells (DACs) we used bidistilled water. The water was obtained from a GFL Glass Bi-Distiller. For the gas-jet during the cryogenic loading we used CO_2 gas as purchased (Nippon gases, purity $\geq$ 99.995%).

1.2. High-pressure experiments

The high-pressure experiments were carried out in Boehler-Almax type DACs equipped with diamonds having an opening angle of 70° and 300 µm sized culets on both sides.[1] We used Re-gaskets, which were pre-indented to thicknesses of $\approx$ 40 µm. Afterwards, sample chambers with $\approx$ 60 µm diameter were drilled into the Re-gaskets using a custom-built laser set-up. The pressure during compression of the DAC was derived from the position of the high frequency edge of the diamond Raman band and we assume an error of at least 5% due to non-hydrostatic conditions in the DAC.[2] In addition, we expect that the pressure conditions in the DAC before laser heating are very likely non-hydrostatic as CO_2-III may sustain pressure gradients up to 0.2 GPa µm^{-1} at high pressures without heating.[3]

In a first step, we added a drop of bidistilled water into the sample chamber of the DAC. Afterwards, we waited until a significant amount was evaporated and closed the DAC tightly. In a second step, the DAC was placed on a liquid nitrogen cooled Cu-holder and cooled down for the cryogenic loading using a custom-built cryogenic loading system (see Spahr *et al.*[4]) derived from an earlier concept developed for similar studies.[5] After reaching $\approx$ 273 K the DAC was opened again. Finally, the DAC was cooled down to $\approx$ 100 K and CO_2-I (dry ice) was directly condensed into the gasket hole from the CO_2 gas jet. We used a small nozzle to direct the CO_2 gas jet with 5 l min^{-1} directly at the gap between the upper diamond and the gasket. The precipitation of the CO_2 in the gasket hole was monitored using an optical microscope and a camera. After a sufficient amount of CO_2 was gathered in the gasket hole, the DAC was tightly closed again.

1.3. Laser heating

The H_2O + CO_2 mixture was laser-heated from both sides at the target pressure of the experiment (40(2) GPa) using a custom-built set-up equipped with a Coherent Diamond K-250 pulsed CO_2 laser ($\lambda = 10600$ nm).[6] The laser power was adjusted to achieve a coupling of the laser to the sample, using a laser power between 1–6 W. The maximum temperature achieved during the laser-heating was $T_{\mathrm{max}} = 1000(300)$ K. The temperatures were determined by the two-color pyrometer method, employing Planck and Wien fits.[7] The heating time during the experiments was $\approx$ 30 min. It is well established that laser-heating in DACs always suffers from large temperature gradients and the actual temperature is strongly dependent on the coupling of the laser with the sample, especially at lower temperatures. At these low temperatures we estimate an uncertainty of at least $\pm$20% of the nominal temperature in the laser-heated region depending on the focus of the laser beam, based on typical 2D temperature-gradient determination experiments performed in DACs.[8]

1.4. Raman spectroscopy

High-pressure Raman spectroscopy was performed in the DACs using an Oxford Instruments WITec alpha 300R Raman imaging microscope. The Raman microscope was equipped with an Olympus SLMPan N 50× objective. The measurements were performed using the 532 nm laser. We employed the 1800 grooves mm^{-1} grating of the WITec UHTS 300S (VIS-NIR) spectrograph in combination with an Andor DR316B-LDC-DD CCD detector for the measurements. The laser power was 100 mW on the sample and the spot size of the Raman laser was $\approx$ 0.8 µm. We assume a depth resolution of $\approx$ 6 µm in the direction of the laser beam. Raman maps were measured on a grid with a step-size of 0.5 µm. The background of the Raman spectra was corrected using the software package Fityk.[9]

*d.spahr@kristall.uni-frankfurt.de

1.5. Single-crystal synchrotron X-ray diffraction

Single-crystal synchrotron X-ray diffraction was carried out at the synchrotron PETRA III (DESY) in Hamburg, Germany, at the Extreme Conditions Beamline P02.2.[10] The beam size on the sample was $\approx 2 \times 2$ µm^2 (FWHM), focused by Kirkpatrick Baez mirrors. The diffraction data were collected using a Perkin Elmer XRD1621 detector, a wavelength of 0.2903 Å (42.7 keV) and a sample to detector distance of 420.5 mm. We rotated the DAC by $\pm 34°$ around the vertical axis perpendicular to the beam while collecting frames in 0.25° steps with 4 s acquisition time per frame.

The sample to detector distance was calibrated using the powder diffraction pattern of a CeO_2 standard in conjunction with the software DIOPTAS.[11] The diffractometer/detector geometry for the analysis of the single crystal diffraction data was calibrated using diffraction data collected from a single crystal of enstatite ($MgSiO_3$) in a DAC at ambient pressure. After the data collection, the reflections were indexed and integrated employing CrysAlis^{PRO} (version 43.67a).[12] We used the Domain Auto Finder program (DAFi) to find possible single-crystal domains for the subsequent data reduction.[13] The structure solution and refinement were performed using the software package OLEX2 employing SHELXT for the crystal structure determination and SHELXL for the refinement.[14,15,16]

1.6. Density functional theory-based calculations

First-principles calculations were carried out within the framework of density functional theory (DFT), employing the Perdew-Burke-Ernzerhof (PBE) exchange-correlation functional and the plane wave/pseudopotential approach implemented in the CASTEP simulation package.[17,18,19] "On the fly" norm-conserving or ultrasoft pseudopotentials generated using the descriptors in the CASTEP data base were employed in conjunction with plane waves up to a kinetic energy cutoff of 1020 eV or 630 eV, for norm-conserving and ultrasoft pseudopotentials, respectively. The accuracy of the pseudopotentials is well established.[20] A correction scheme for van der Waals (v.d.W.) interactions was applied in the DFT-calculations. We employed the correction scheme developed by Tkatchenko and Scheffler.[21] A Monkhorst-Pack grid was used for Brillouin zone integrations.[22] We used a distance between grid points of <0.023 Å^{-1}. Convergence criteria for geometry optimization included an energy change of $<5 \times 10^{-6}$ eV atom^{-1} between steps, a maximal force of <0.008 eV Å^{-1} and a maximal component of the stress tensor <0.02 GPa. Phonon frequencies were obtained from density functional perturbation theory (DFPT) calculations.[23,24] Raman intensities were computed using DFPT with the "$2n+1$" theorem approach.[25] All DFPT calculations were carried out with norm-conserving pseudopotentials. Calculations with ultrasoft potentials were employed for p,V-calculations.

2. Supplementary Discussion

2.1. *Single-crystal synchrotron X-ray diffraction*

In order to determine the crystal structure of the unknown phase, we employed synchrotron X-ray diffraction in the area of the sample chamber where we observed mainly Raman modes of the unknown phase. We collected X-ray diffraction data on a grid using a spot size of $\approx 2 \times 2\ \mu m^2$ in order to locate promising positions for the collection of single-crystal diffraction data. Afterwards, we collected diffraction data suitable for single-crystal X-ray diffraction analysis on different positions with long exposure times (16 s/°). The reciprocal space reconstruction for the ($4kl$) plane of the crystal on which the crystal structure was determined is shown in Fig. S 1 a, demonstrating the high quality of the collected diffraction data.

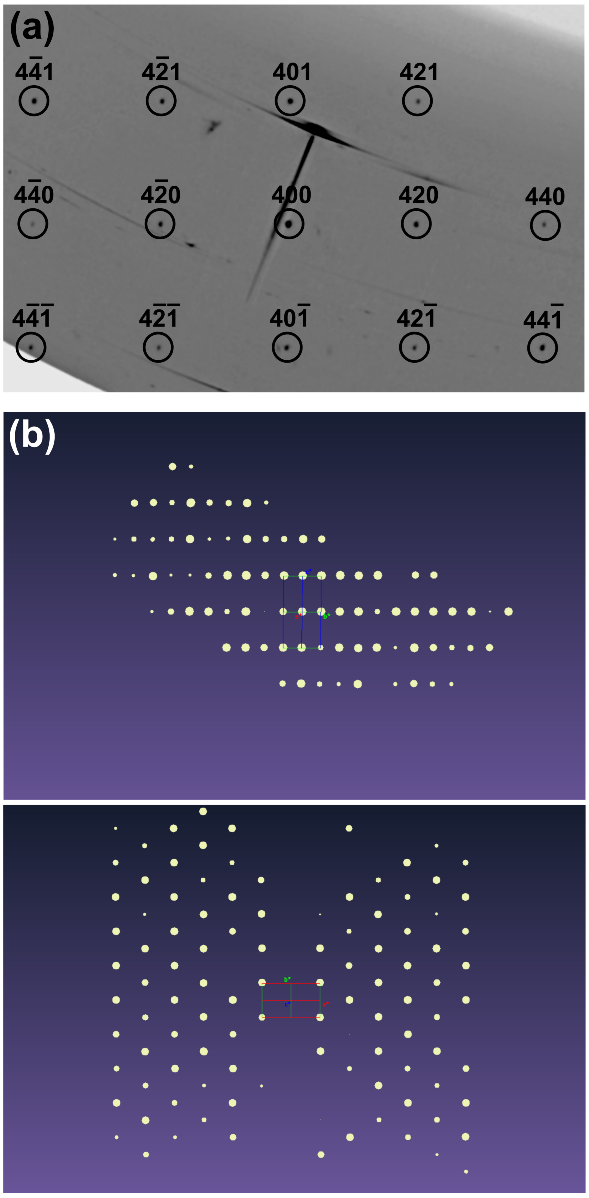

Figure S 1: (a) Reciprocal space reconstruction for the ($4kl$) plane. (b) Schematic depiction of the reflections in reciprocal space in the Ewald-Explorer in CrysAlis after data reduction which were used for the refinement. Projection of the reciprocal space are shown along a^* (top) and c^* (bottom).

In addition to the reflection of the unknown phase, we observed reflections and powder rings of different CO_2 phases (III and V) and of the diamonds in the diffraction data (Fig. S 1 a). Fig. S 1 b shows the projection of the reciprocal space along a^* (top) and c^* (bottom). The effect of the shading of diffracted beams due to the metallic body of the DAC can be observed.

Table S 1: Structural parameters of H_2CO_3-$Cmc2_1$ at 40(2) GPa from single-crystal structure solution (ambient temperature) in comparison to data from DFT calculations (athermal limit).

	**Single Crystal**	**DFT**
**Crystal data**		
Crystal system	Orthorhombic	Orthorhombic
Space group	$Cmc2_1$	$Cmc2_1$
Chemical formula	H_2CO_3	H_2CO_3
M_r	62.03	62.03
a (Å)	7.286(3)	7.5010
b (Å)	4.2747(10)	4.2068
c (Å)	3.809(4)	3.8780
α (°)	90.0	90.0
β (°)	90.0	90.0
γ (°)	90.0	90.0
V (Å^3)	118.63(12)	122.37
Z	4	4
**Data collection**		-
F_{000}	128	-
θ range (°)	2.23–17.09	-
measured reflections	251	-
independent reflections	137	-
reflections $I > 2\sigma(I)$	121	-
R_{int}	0.014	-
		-
**Refinement**		-
$R_1[I > 2\sigma(I)]$, $wR_2(I)$	0.046, 0.112	-
No. of reflections	137	-
No. of parameters	21	-
No. of restraints	2	-
No. of constraints	1	-
$\Delta\rho_{\max}$, $\Delta\rho_{\min}$ (e Å^{-3})	0.36, −0.42	-

We solved the crystal structure of the unknown phase in space group $Cmc2_1$ (No. 36) with $Z = 4$ and H_2CO_3 composition. The structural model is identical with the one predicted earlier by evolutionary algorithm in the C-H-O ternary system for H_2CO_3.[26] The low R_1-value of (4.6%) reveals a reasonable structure refinement. The reflection to parameter ratio is acceptable (6.5:1). The displacement parameters of the carbon and the oxygen atoms were refined anisotropically. In order to reduce the number of free parameters the displacement parameters of the two oxygen atoms were constrained to be identical. The displacement parameter of the hydrogen atom was refined isotropically and the hydrogen position was refined with a "riding model" and $U_{iso}(H) = 1.5U_{iso}(O)$. The O–H bond was allowed to rotate and a soft constraint for the H–O bond distances (0.90 Å with ± 0.05 Å) has been introduced. The very low R_{int} (1.4%) in combination with

the high $F^2/\sigma(F^2)$-value ($\approx$ 90 up to 0.8 Å^{-1} and $\approx$ 40 up to 0.5 Å^{-1}) reveal a very high quality of the experimental data and of the data reduction, taken into account that only light elements (H, C, O) were present in the crystal structure and the diffraction experiment was performed on a multi-grain reaction product inside a DAC at high pressure. Table 1 lists the experimental crystallographic parameters of H_2CO_3-*Cmc*2$_1$ at 40(2) GPa in comparison to data derived from the DFT calculations. The atomic coordinates are listed in Table S 2. The anisotropic displacement parameters of the the carbon and the oxygen atoms can be found in the cif-file.

Table S 2: Atomic coordinates and equivalent/isotropic displacement parameters (Å^2) of H_2CO_3-*Cmc*2$_1$ at 40(2) GPa obtained by single crystal structure refinement (1st line) and from DFT-based calculations (2nd line).

Atom	Site	x	y	z	U_{iso}/U^*_{eq}
H1	8*b*	0.351(8)	0.09(1)	0.30(2)	0.015
		0.3379	-0.0027	0.3214	
C1	4*a*	1/2	0.3349(8)	0.581(2)	0.010(2)
		1/2	0.3361	0.5812	
O1	8*b*	0.3465(3)	0.1761(4)	0.502(2)	0.011(1)
		0.3489	0.1769	0.5016	
O2	4*a*	1/2	0.6304(5)	0.4385(2)	0.011(1)
		1/2	0.6300	0.4402	

* U_{eq} is derived as 1/3 of the orthogonalized U_{ij} tensor

2.2. *H_2O content in the sample chamber*

In order to confirm the presence of H_2O in the sample chamber of the DAC after the cryogenic loading, we used Raman spectroscopy (Fig. S 2). We observed a weak Raman signal of H_2O-VII at high wavenumbers ($\approx$ 3100 cm^{-1}) after the loading at $\approx$ 10 GPa, in agreement with earlier Raman studies.[27,28] In addition, we observed the strong characteristic Raman modes of CO_2-I at low wavenumbers ($<$ 300 cm^{-1}).[29]

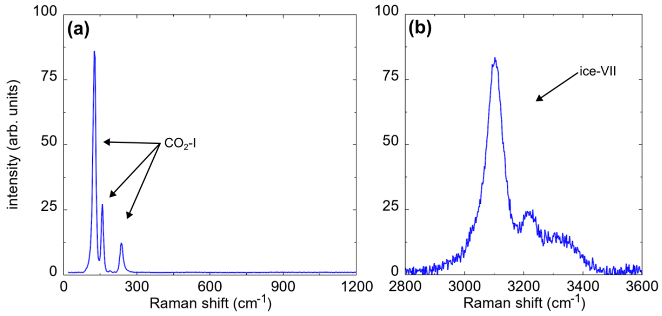

Figure S 2: Raman spectra of (a) CO_2-I and (b) H_2O-VII in the sample chamber of the DAC at $\approx$ 10 GPa after the cryogenic loading.

2.3. *Raman spectroscopy at 40(2) GPa*

We are aware, that the position of the strongest Raman mode of CO_2-V ($\approx$ 810 cm^{-1}) is very close to one of the dominant Raman modes of H_2CO_3-*Cmc*2$_1$ ($\approx$ 820 cm^{-1}). At first sight they could be mixed up. A closer inspection of the peak positions and the distribution of the corresponding phases in the sample chamber of the DAC clearly shows that the Raman mode at $\approx$ 820 cm^{-1} corresponds to H_2CO_3-*Cmc*2$_1$. The 2D-Raman map of the peak at $\approx$ 910 cm^{-1}, which unequivocally belongs to H_2CO_3-*Cmc*2$_1$ and the one for the mode at $\approx$ 820 cm^{-1} are in good agreement (Fig. S 3).

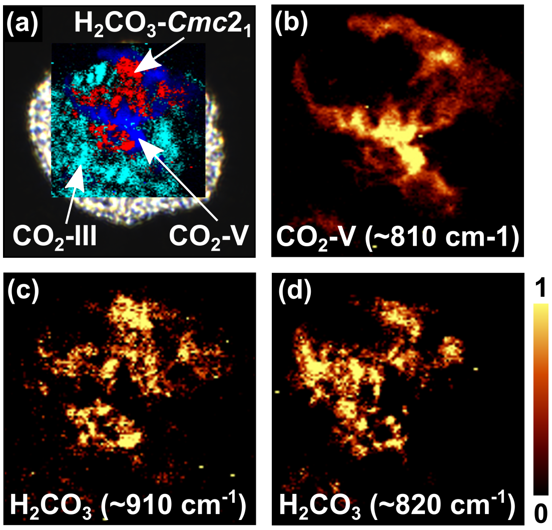

Figure S 3: (a) 2D-Raman map showing the distribution of H_2CO_3-*Cmc*2$_1$, CO_2-III and CO_2-V overlayed on a picture of the sample chamber after laser heating at 40(2) GPa. Raman map of: (b) CO_2-V ($\approx$ 810 cm^{-1}), (c) H_2CO_3-*Cmc*2$_1$ ($\approx$ 910 cm^{-1}) and (d) H_2CO_3-*Cmc*2$_1$ ($\approx$ 820 cm^{-1}).

2.4. *Compressibility of H_2CO_3-Cmc2$_1$*

We used our DFT-based calculations to derive the p, V relation for H_2CO_3-*Cmc*2$_1$ in the pressure range between 30 GPa and 90 GPa. The calculated p, V-data were fitted with a 3rd-order Birch-Murnaghan equation of states (EoS) in order to determine the bulk modulus (K_0) using the software package EOSFit7-GUI (Fig. S 4).[30,31,32]

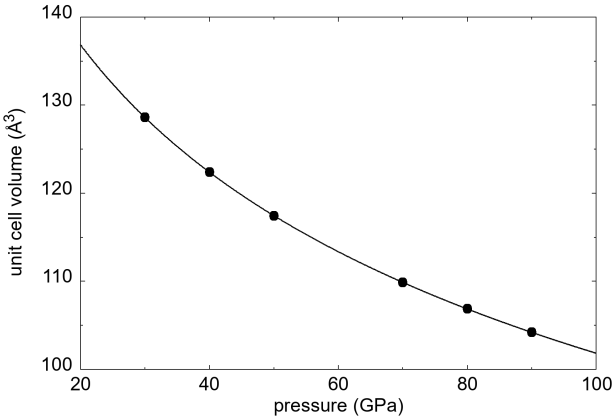

Figure S 4: A Birch-Murnaghan EoS was fitted to the p, V-data of H_2CO_3-*Cmc*2$_1$ obtained by DFT-based calculations in the pressure range between 30 GPa and 90 GPa.

The theoretical bulk modulus and its derivative of polymerized H_2CO_3-*Cmc*2$_1$ derived from the EoS-fit in the

pressure range between 30–90 GPa are $K_0 = 50(2)$ GPa with $K_p = 5.2(1)$. The obtained bulk modulus is significantly higher than the one derived for the unpolymerized polymorph H_2CO_3-$P2_1/n$ ($K_0 = 14.2(4)$ GPa with $K_p = 6.1(1)$) synthesized at significantly lower pressures (≤ 10 GPa).[33] We found that the compression behavior is significantly anisotropic. Between 30 GPa and 90 GPa the compression along the *a*-axis is $\approx 10\%$, while it is only $\approx 6\%$ along the *b*- and *c*-axes in the same pressure range.

2.5. Hydrogen bonding in H_2CO_3-$Cmc2_1$

The anisotropic compression behavior is mainly caused by orientation of the $[CO_4]^{4-}$-tetrahedra chains along the *c*-axis and by the hydrogen bonding arrangement in H_2CO_3-$Cmc2_1$. There is a network of strong O–H$\cdots$O bonds in the crystal structure in the b, c-plane (Fig. S 5). The O–H$\cdots$O distance is ≈ 1.45 Å. In contrast the O–H$\cdots$O distance between the chains along the *c*-axis is significantly larger (≈ 2.31 Å).

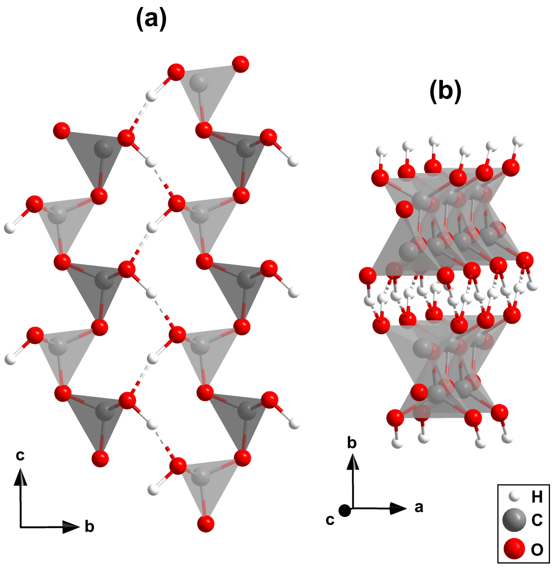

Figure S 5: Cystal structure of H_2CO_3-$Cmc2_1$ from DFT calculations at 40 GPa (a) along the *a*-axes and (b) along the *c*-axes. Short O–H$\cdots$O distances (≈ 1.45 Å) are shown by dashed lines.

2.6. DFPT calculations on H_2CO_3 and CO_2

We used our DFPT calculations to obtain the Raman frequencies and intensities for CO_2-III (*Cmca*). The results are listed in Table S 3.

Table S 3: Raman shifts (cm^{-1}) and Raman activity (Å^4/amu) from DFT-based calculations for CO_2-III (*Cmca*) at 45 GPa.

Raman shift	Raman activity
362	22.9
377	24.4
383	2.3
431	16.2
1401	351.1
1406	60.2

In addition, we calculated the frequencies and intensities for the high pressure polymorph CO_2-V ($I\bar{4}2d$). The results are listed in Table S 4.

Table S 4: Raman shifts (cm^{-1}) and Raman activity (Å^4/amu) from DFT-based calculations for CO_2-V ($I\bar{4}2d$) at 45 GPa.

Raman shift	Raman activity
464	8.5
464	8.5
756	12.8
793	108.7
881	9.9
881	9.9
890	5.8
1046	0.5
1046	0.5
1047	18.7
1190	4.0
1190	4.0
1221	1.4

Table S 5 lists the Raman frequencies and intensities for H_2CO_3-$Cmc2_1$ from the DFPT calculations.

Table S 5: Raman shifts (cm^{-1}) and Raman activity (Å^4/amu) from DFT-based calculations for H_2CO_3 ($Cmc2_1$) at 40 GPa.

Raman shift	Raman activity	Raman shift	Raman activity
320	2.3	1047	19.8
349	0.0	1050	0.0
352	4.9	1068	0.9
367	0.1	1144	2.4
396	15.1	1148	0.2
562	23.6	1180	0.1
579	9.5	1195	10.6
617	1.7	1248	0.7
644	2.0	1391	0.0
647	5.3	1430	37.8
727	6.0	1452	48.5
734	27.1	1603	28.0
811	95.0	2520	1446.6
853	3.5	2524	18.1
859	0.2	2880	28.1
915	79.7	2914	403.6
988	0.3		

References

(1) Boehler, R. New diamond cell for single–crystal X-ray diffraction. *Rev. Sci. Instrum.* **2006**, *77*, 115103–1–115103–3, DOI: 10.1029/JB091iB05p04673

(2) Akahama, Y.; Kawamura, H.; Pressure calibration of diamond anvil Raman gauge to 310 GPa. *J. Appl. Phys.* **2006**, *100*, 043516, DOI: 10.1063/1.2335683

(3) Yoo, C. S.; Cynn, H.; Gygi, F.; Galli, G.; Iota, V.; Nicol, M.; Carlson, S.; Häusermann, D.; Mailhiot, C. Crystal Structure of Carbon Dioxide at High Pressure: "Superhard" Polymeric Carbon Dioxide. *Phys. Rev. Lett.* **1999**, *83*, 5527–5530, DOI: 10.1103/PhysRevLett.83.5527

(4) Spahr, D.; König, J.; Bayarjargal, L.; Luchitskaia, R.; Milman, V.; Perlov, A.; Liermann, H.-P.; Winkler, B.; Synthesis and Structure of Pb[C_2O_5]: An Inorganic Pyrocarbonate Salt. *Inorg. Chem.* **2022**, *61*, 9855–9859, DOI: 10.1021/acs.inorgchem.2c01507

(5) Scelta, D.; Ceppatelli, M.; Ballerini, R.; Hajeb, A.; Peruzzini, M.; Bini, R. Sprayloading: A cryogenic deposition method for diamond anvil cell. *Rev. Sci. Instrum.* **2018**, *89*, 053903, DOI: 10.1063/1.5011286

(6) Bayarjargal, L.; Fruhner, C.-J.; Schrodt, N.; Winkler, B. $CaCO_3$ phase diagram studied with Raman spectroscopy at pressures up to 50 GPa and high temperatures and DFT modeling. *Phys. Earth Planet. Inter.* **2018**, *281*, 31–45, DOI: 10.1016/j.pepi.2018.05.002

(7) Benedetti, L. R.; Loubeyre, P. Temperature gradients, wavelength-dependent emissivity, and accuracy of high and very-high temperatures measured in the laser-heated diamond cell. *High Press. Res.* **2004**, *24*, 423–455, DOI: 10.1080/08957950412331331718

(8) Du, Z.; Amulele, G.; Benedetti, L. R.; Lee, K. K. M. Mapping temperatures and temperature gradients during flash heating in a diamond-anvil cell. *Rev. Sci. Instrum.* **2013**, *84*, 075111, DOI: 10.1063/1.4813704

(9) Wojdyr, M. *Fityk*: a general-purpose peak fitting program. *J. Appl. Cryst.* **2010**, *43*, 1126–1128, DOI: 10.1107/S0021889810030499

(10) Liermann, H.-P.; Konôpková, Z.; Morgenroth, W.; Glazyrin, K.; Bednarčik, J.; McBride, E. E.; Petitgirard, S.; Delitz, J. T.; Wendt, M.; Bican, Y.; Ehnes, A.; Schwark, I.; Rothkirch, A.; Tischer, M.; Heuer, J.; Schulte-Schrepping, H.; Kracht, T.; Franz, H. The Extreme Conditions Beamline P02.2 and the Extreme Conditions Science Infrastructure at PETRAIII. *J. Synchrotron Radiat.* **2014**, *22*, 908–924, DOI: 10.1107/S1600577515005937

(11) Prescher, C.; Prakapenka, V. B. *DIOPTAS*: a program for reduction of two-dimensional X-ray diffraction data and data exploration. *High. Press. Res.* **2015**, *35*, 223–230, DOI: 10.1080/08957959.2015.1059835

(12) Agilent, CrysAlis PRO, Yarnton, England, **2014**

(13) Aslandukov, A.; Aslandukov, M.; Dubrovinskaia, N.; Dubrovinsky, L. *Domain Auto Finder* (*DAFi*) program: the analysis of single-crystal X-ray diffraction data from polycrystalline sample. *J. Appl. Cryst.* **2022**, *55*, 1383–1391, DOI: 10.1107/S1600576722008081

(14) Dolomanov, O. V.; Bourhis, L. J.; Gildea, R. J.; Howard, J. A. K.; Puschmann, H. *OLEX2*: a complete structure solution, refinement and analysis program. *J. Appl. Cryst.* **2009**, *42*, 339–341, DOI: 10.1107/S0021889808042726

(15) Sheldrick, G. M. *SHELXT* — Integrated space-group and crystal-structure determination. *Acta. Cryst.* **2015**, *A71*, 3–8, DOI: 10.1107/S2053273314026370

(16) Sheldrick, G. M. Crystal structure refinement with *SHELXL*. *Acta. Cryst.* **2015**, *C71*, 3–8, DOI: 10.1107/S2053229614024218

(17) Hohenberg, P.; Kohn, W. Inhomogeneous Electron Gas. *Phys. Rev.* **1967**, *136*, B864–B871, DOI: 10.1103/PhysRev.136.B864

(18) Perdew, J. P.; Burke, K.; Ernzerhof, M. Generalized Gradient Approximation Made Simple. *Phys. Rev. Lett.* **1996**, *77*, 3865–3868, DOI: 10.1103/PhysRevLett.77.3865

(19) Clark, S. J.; Segall, M. D.; Pickard, C. J.; Hasnip, P. J.; Probert, M. I. J.; Refson, K.; Payne, M. C. First principles methods using CASTEP. *Z. Kristallogr.* **2005**, *220*, 567–570, DOI: 10.1524/zkri.220.5.567.65075

(20) Lejaeghere, K.; Bihlmayer, G.; Björkman, T.; Blaha, P.; Blügel, S.; Blum, V.; Caliste, D.; Castelli, I. E.; Clark, S. J.; Dal Corso, A. et al. Reproducibility in density functional theory calculations of solids. *Science* **2016**, *351*, aad3000, DOI: 10.1126/science.aad3000

(21) Tkatchenko, A.; Scheffler, M. Accurate Molecular Van Der Waals Interactions from Ground-State Electron Density and Free-Atom Reference Data. *Phys. Rev. Lett.* **2009**, *102*, 073005, DOI: 10.1103/PhysRevLett.102.073005

(22) Monkhorst, H. J.; Pack, J. D. Special points for Brillouin-zone integrations. *Phys. Rev. B* **1976**, *13*, 5188–5192, DOI: 10.1103/PhysRevB.13.5188

(23) Baroni, S.; de Gironcoli, S.; Dal Corso, A.; Giannozzi, P. Phonons and related crystal properties from density-functional perturbation theory. *Rev. Mod. Phys.* **2001**, *73*, 515–562, DOI: 10.1103/RevModPhys.73.515

(24) Refson, K.; Tulip, P. R.; Clark, S. J. Variational density-functional perturbation theory for dielectrics and lattice dynamics. *Phys. Rev. B* **2006**, *73*, 155114, DOI: 10.1103/PhysRevB.73.155114

(25) Miwa, K. Prediction of Raman spectra with ultrasoft pseudopotentials. *Phys. Rev. B* **2011**, *84*, 094304, DOI: 10.1103/PhysRevB.84.094304

(26) Saleh, G.; Oganov, A. R. Novel Stable Compounds in the C-H-O Ternary System at High Pressure. *Sci. Rep.* **2016**, *6*, 32486, DOI: 10.1038/srep32486

(27) Pruzan, P.; Chervin, J. C.; Gauthier, M. Raman Spectroscopy Investigation of Ice VII and Deuterated Ice VII to 40 GPa. Disorder in Ice VII. *EPL* **1990**, *13*, 81–87, DOI: 10.1209/0295-5075/13/1/014

(28) Hsieh, W.-P.; Chien, Y.-H. High pressure Raman spectroscopy of H_2O-CH_3OH mixtures. *Sci. Rep.* **2015**, *5*, 8532, DOI: 10.1038/srep08532

(29) Olijnyk, H.; Jephcoat, A. P. Vibrational studies on CO_2 up to 40 GPa by Raman spectroscopy at room temperature. *Phys. Rev. B* **1998**, *57*, 879–888, DOI: 10.1103/PhysRevB.57.879

(30) Murnaghan, F. The Compressibility of Media under Extreme Pressures. *Proc. Natl. Acad. Sci.* **1944**, *30*, 244–247, DOI: 10.1073/pnas.30.9.244

(31) Birch, F. Finite Elastic Strain of Cubic Crystals. *Phys. Rev.* **1947**, *71*, 809–824, DOI: 10.1103/PhysRev.71.809

(32) Gonzalez-Platas, J.; Alvaro, M.; Nestola, F.; Angel, R. *EosFit7-GUI*: a new graphical user interface for equation of state calculations, analyses and teaching. *J. Appl. Cryst.* **2016**, *49*, 1377–1382, DOI: 10.1107/S1600576716008050

(33) Spahr, D.; Bykova, E.; Bayarjargal, L.; Bykov, M.; Brüning, L.; Kovalev, V.; Milman, V.; Giordano, N.; Liermann, H.-P.; Winkler, B. Crystal Structure of Carbonic Acid (H_2CO_3) at Elevated Pressures from Single Crystal Diffraction. *Chem. Eur. J.* **2025**, e202501964, DOI: 10.1002/chem.202501964
